# Supplementary material for: Complex Presentation of Hao-Fountain Syndrome Solved by Exome Sequencing Highlighting Co-Occurring Genomic Variants
Source: Genes (Basel). 2022 May 16;13(5):889. doi: 10.3390/genes13050889 (PMC9141324; doi:10.3390/genes13050889)
Supplement: Supplementary file 1 [file genes-13-00889-s001.zip › genes-1700576-supplementary.pdf]

Supplemental Table S1. WES statistics and data output.

WES enrichment kit	SureSelect Human All Exon V7
Sequencing platform	Illumina NovaSeq6000
Target regions coverage >10x	95%
Target regions coverage >20x	94%
Average depth on target	147x
Total number of high-quality variants	72,910
Variants with effect on CDS or affecting splice sites[1]	13,847
Private, clinically associated and low frequency variants[2]	242
Putative disease associated genes[3]	12[4]
Disease genes with pathogenic variants	*USP7*, *PKD2, CFTR*[5]

[1] High-quality non-synonymous SNV plus indels within coding exons and splice regions (-3/+8 nt).

[2] High-quality, rare/private, functionally relevant variants (gnomAD MAF <0.1%; in house database MAF <1%).

[3] High-quality, rare/private, functionally relevant variants with CADD phred>20.0, M-CAP>0.025, either autosomal dominant or recessive disease associated genes.

[4] *ABCC11* (c.1369delC, p.Gln457fs), *CLCN6* (c.668G>A, p.Arg223Gln), *FLNB* (c.731C>T, p.Pro244Leu), *IMPG2* (c.2716C>T, p.Arg906*), *KCNQ3* (c.1918G>A, p.Val640Met), *MLH3* (c.278G>A, p.Arg93Gln), *PKD2* (c.295G>T, p.Glu99*), *SETD1B* (c.4859C>T, p.Pro1620Leu), *SIX5* (c.1288C>G, p.Pro430Ala), *SYNE1* (c.19919A>T, p.His6640Leu), *TMEM43* (c.203T>C, p.Leu68Pro), *USP7* (c.1639G>T, p.Glu547*).

[5] The *CFTR* variants were detected by manual inspection of reads mapping (c.1521_1523delCTT (Phe508Del, rs113993960, VCV000634837], c.1210-12T(5) [IVS8-5T, rs1805177, VCV000242535]).
